# Effectiveness of Nutritional Interventions in Pre-Frail or Frail Older Adults: A Scoping Review

**DOI:** 10.3390/jcm15020604

**Published:** 2026-01-12

**Authors:** Izolde Bouloukaki, Antonios Christodoulakis, Antonia Aravantinou-Karlatou, Dimitrios Vavoulas, Sevasti Peraki, Ioanna Tsiligianni

**Affiliations:** 1 Department of Social Medicine, School of Medicine, University of Crete, 71500 Heraklion, Greece; i.bouloukaki@uoc.gr (I.B.); christodoulakisa@uoc.gr (A.C.); dk.vavoulas@gmail.com (D.V.); sevastiperaki@gmail.com (S.P.); i.tsiligianni@uoc.gr (I.T.); 2 Department of Nursing, School of Health Sciences, Hellenic Mediterranean University, 71410 Heraklion, Greece; 3 Department of Primary Care and Population Health, Medical School, University of Nicosia, 2417 Nicosia, Cyprus

**Keywords:** nutritional interventions, frailty, pre-frailty, older adults, scoping review

## Abstract

**Background/Objectives:** Frailty is a complex geriatric syndrome that requires a comprehensive evaluation. This approach should consider not only physical symptoms but also cognitive, psychological, functional, nutritional, and social aspects. Nutritional status is a critical and modifiable factor, making it a key area for intervention in the prevention and management of frailty. Therefore, a scoping review was conducted to determine the effectiveness of nutritional intervention in frail/pre-frail older adults. **Methods:** We adopted the PRISMA-Scoping approach, and we sought studies published in MEDLINE and Scopus databases. **Results:** We screened 3038 studies and finally we included a total of 19 studies. The interventions included nutritional education, counseling, and supplementation, most in combination with exercise. Nutritional interventions improved frailty status, physical performance, functional status, and quality of life in older adults. **Conclusions:** Consequently, this scoping review indicates that multifactorial interventions combining nutritional interventions with physical activity are clearly more effective than a single approach in improving the quality of life of older patients with frailty. These provisional findings may generate hypotheses for policymakers to consider multifaceted interventions for older patients with frailty.

## 1. Introduction

With improvements in social and economic progress and health facilities, mortality rates have declined, especially for the elderly [1]. However, individuals over the age of 65 years are presumably at heightened risk of multiple chronic conditions, reduced physiological reserves, cognitive and functional impairments, and frailty [2,3]. Frailty is a complex geriatric syndrome that may affect more than half of older adults in the community worldwide, depending on the classification criteria and diagnostic tools used [3,4,5,6]. It significantly impairs physical and functional capacity, posing major challenges for healthcare systems worldwide [7,8]. This geriatric condition makes older people more susceptible to adverse health effects and increases the risk of functional inability, immobility, falls, loneliness, social isolation, disability, hospitalization, institutionalization, mortality, and healthcare costs [1,2,3].

As defined by Fried et al., frailty is a clinical syndrome characterized by the presence of three or more of five specific criteria: (1) unintentional weight loss (10 lbs. in the past year), (2) self-reported exhaustion, (3) weakness (reduced grip strength), (4) slow walking speed, and (5) low physical activity [9]. Individuals meeting one or two of these conditions are classified as “pre-frail” [9]. Using the Integrated Care for Older People (ICOPE) framework, the World Health Organization has recently conceptualized frailty, expanding it beyond physical decline to encompass a progressive loss of “intrinsic capacity” [10]. “Intrinsic capacity” refers to mental function, emotional state, physical movement, sensory perception, and the ability to engage socially and contribute to society. From this multifaceted perspective, frailty emerges as a complex and dynamic condition that requires a comprehensive evaluation. This approach should consider not only physical symptoms but also cognitive, psychological, functional, nutritional, and social aspects [11,12].

Even though frailty is generally considered to be a complex multifactorial process; studies highlight that poor nutritional status is a key component in the development of frailty in older adults [8,13,14,15,16], affecting all five criteria in Fried’s frailty definition [17]. The nutritional status and nutrient intake of older adults are influenced by several factors related to the aging process (i.e., slower metabolism and muscle loss) [18]. Frail individuals are five times more prone to malnutrition than those who are not frail, and addressing malnutrition could prevent approximately 2.5–5.0% of frailty cases [19]. Studies have also highlighted the protective role of healthy dietary patterns against frailty [20,21]. High adherence to the Mediterranean diet, high consumption of fruits and vegetables, and adequate micronutrient and mineral intake according to the age and the condition of each person are positively associated with lower risk of frailty onset and progression [22,23,24]. As such, nutritional status is a critical and modifiable factor, making it a key area for intervention in the prevention and management of frailty [25,26,27].

During the past two decades, there has been a significant rise in research regarding the association between nutritional status and frailty in older individuals, and nutritional interventions are receiving more recognition as a potential approach to better manage and potentially even reverse frailty [28]. Several studies have examined the effect of nutritional interventions on frailty, yet the evidence is difficult to implement in clinical practice [29,30,31,32,33,34,35,36]. Furthermore, the effect of nutritional interventions on older individuals in a pre-frail state is uncertain [37]. Available systematic reviews highlight the scarcity of evidence on the effect of nutritional interventions on a diverse range of outcomes in frail and pre-frail older adults [21], making it difficult to evaluate their effectiveness. With the potential of appropriate interventions to prevent, postpone, or reverse frailty, the varied outcomes of previous studies and the range of intervention approaches present obstacles to healthcare professionals and policymakers in ensuring effective implementation [38,39]. For these reasons, a scoping review, utilizing current evidence to broadly map the heterogeneous evidence on the effectiveness of nutritional interventions, is essential. Therefore, the objective of this review was to identify and highlight existing data on nutritional interventions for older adults with pre-frailty and frailty with the potential to identify and prioritize gaps in the development of holistic care plans designed to manage the complex aspects of frailty.

## 2. Materials and Methods

### 2.1. Search Strategy

This scoping review followed the Joanna Brigg’s Institute guidelines for scoping reviews [40], and the findings were reported using the PRISMA-ScR checklist (Supplementary Materials Table S1) [41]. The research protocol was retrospectively registered (protocol number: INPLASY2025120031) on the International Platform of Registered Systematic Review and Meta-analysis Protocols (INPLASY) at https://doi.org/10.37766/inplasy2025.12.0031 in December 2025, accessed on 9 December 2025. It should be noted that the protocol’s methodologies were consistent from conception to registration.

### 2.2. Inclusion and Exclusion Criteria

We included original peer-reviewed published studies, like randomized and non-randomized controlled trials, cohort studies, and case–control studies investigating the effects of nutritional interventions on frail and pre-frail older adults. Specifically, studies had to meet the following criteria to be considered for inclusion in this review: studies with available full texts, published in English and the last ten years, including individuals aged 60 years and above, individuals who were diagnosed either with frail or pre-frailty, community-dwelling older adults, adults who reside in nursing homes, outpatients of geriatric clinics, or inpatients in acute care wards. We excluded review studies, case reports, commentaries, editorials, letters, conference abstracts, book chapters, and studies published in languages other than English.

In terms of types of interventions, this review included studies that utilized at least one of the following nutritional interventions: nutritional education/dietary prescription, the employment of hypercaloric or hyperproteic dietary oral supplements, or the implementation of specific diets. Additionally, we considered studies that combined the aforementioned interventions with another intervention, whether single or multifactorial. The outcome measurements in this study included frailty score, dietary intake outcomes and nutritional knowledge, physical performance outcomes, functional outcomes, psychological outcomes, cognitive function, quality of life, and mortality.

### 2.3. Information Sources and Search Strategy

The search of the literature was undertaken from July to August 2025, using the electronic biomedical databases MEDLINE and Scopus. We used variations of the following related keywords, “Frailty”, “Nutritional”, “nutrition”, “Interventions”, “Older patients”, and “pre-Frailty”, “Frailty” AND “nutritional interventions”, “elderly patients with frailty”, “nutritional interventions in elderly patients with frailty”; these were also screened for all known variations and synonyms, with more comprehensive inclusion as needed. Specifically, the full search string for both data bases was “Frailty” OR “Pre-frailty” AND “Nutritional Interventions” OR “nutrition Interventions” AND “Older adults” OR “elderly”, with the following filters, 2015–2025 and English language. Due to the exploratory nature of this scoping review, which sought to map the extent of evidence rather than assess its efficacy, no formal quality appraisal was conducted.

In this scoping review, we analyzed the data regarding the effectiveness of nutritional interventions for the management of pre-frailty and frailty in older adults. Four independent researchers conducted an examination of the reference lists derived from electronic searches. Additionally, they manually searched the reference lists of pertinent publications, encompassing review articles on frailty and original studies deemed appropriate for the review. After removing duplicates, they evaluated each extraction form and discussed any discrepancies in a thorough appraisal process. The extracted data contained details about the studies, including the time period when the study was conducted; geographical location; study design; types of interventions and comparators; duration of the intervention and of follow-up; inclusion/exclusion criteria; sample size; characteristics of the population; presence of simultaneous interventions; diagnostic criteria of frailty; nutritional interventions; details of the intervention, including type, dose, frequency, and duration; control treatment; outcome measures; conclusions reported by the study authors; and research limitations. Afterwards, they performed data extraction to guarantee accuracy and consistency. All disagreements during the review’s inclusion phase were resolved through discussion to reach a consensus. In instances where reviewer consensus was not achieved, a fifth, independent reviewer was employed to solve it.

## 3. Results

### 3.1. Screening and Procedure

**Figure 1:**
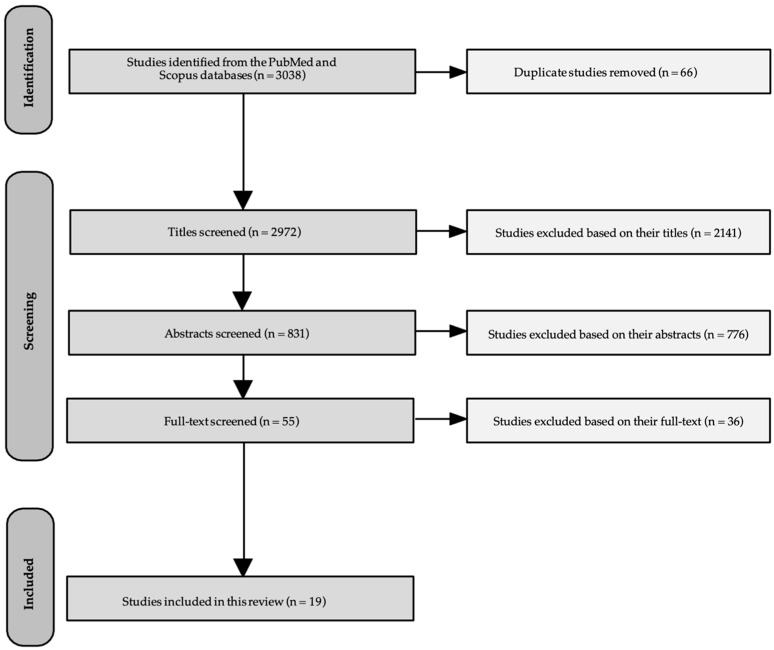
PRISMA flow diagram for this scoping review.

The database initially included 3038 articles for our scoping review. Following the first screening, the duplicates (n = 66) were removed, and the remaining 2972 articles were screened based on their titles, discerning those referring to nutritional interventions. Subsequently, 831 titles met the inclusion criteria, designating the articles eligible for a second screening phase based on relevance to our aim and study design, as described in their abstract texts. During that procedure, a second/further evaluation was conducted on 55 abstracts that satisfied the inclusion criteria, and their full texts were obtained to be further screened. However, 36 studies were excluded in accordance with the criteria for inclusion/exclusion, as described in the methodology. Consequently, 19 full-text articles were identified to meet the inclusion criteria and were included in the current scoping review. The PRISMA flow diagram summarizing this process is presented in Figure 1.

### 3.2. Overview Characteristics of the Included Studies

The characteristics of the 19 included studies [36,42,43,44,45,46,47,48,49,50,51,52,53,54,55,56,57,58,59] are presented in Table 1. The majority of the studies (14/19) were categorized as randomized controlled trials [36,43,44,45,47,49,50,51,52,53,54,55,56,58]. The included studies were conducted globally, included five studies from Spain [43,44,45,50,56], two studies from the USA [57,58], two studies from Taiwan [46,47], two studies from Canada [52,55], and one study from Singapore [54], Japan [53], Portugal [42], Australia [36], Ethiopia [48], China [49], and Denmark [51]. Overall, 13/19 studies were conducted in community and home care settings [42,45,48,49,50,51,52,53,54,55,57,58,59]. The studies were published from 2015 to 2025. Regarding the sample size, it ranged from 13 to 821 older patients, with 10 studies [42,43,44,48,49,51,52,53,57,59] conductedon frail older patients and 9 studies [36,45,46,47,50,54,55,56,58] conducted on frail/pre-frail older patients. For the control group, the majority of studies (12 out of 19 studies) used standard care practices [36,43,44,45,47,50,51,53,54,55,56,57]. The investigation’s intervention period ranged from a minimum of 4 weeks to a maximum of 40 weeks. The most common outcomes assessed were frailty status (14/19 studies) [36,42,43,45,46,47,48,49,50,53,54,56,57,59], functional status (12/19 studies) [36,43,44,45,46,48,50,52,53,56,57,58], and physical performance (12/19 studies) [36,44,46,47,49,53,54,55,56,57,58,59].

### 3.3. Instruments

All the included studies used assessment instruments for identifying frailty. Ten studies employed Fried’s Frailty Phenotype (FP) Scale [42,43,44,45,47,49,50,56,57], while three studies utilized the CHS physical frailty criteria [54,55,58]. Additionally, eight studies used a dynamometer to evaluate the handgrip strength [36,44,47,49,50,55,56,57] and three studies used the Barthel index for activities of daily living and functional status [43,44,45]. To assess nutritional, cognitive, and functional health, along with physical performance, a diverse set of assessment tools were employed (Table 2).

### 3.4. Intervention Characteristics

Of the nutritional interventions, 6/19 studies included nutritional counseling [43,46,47,51,57,58], 8/19 nutritional education [45,48,49,50,52,53,55,59], and 5/19 nutritional supplementation [42,44,47,54,56] (Table 1); these were frequently enriched by technology (4/19) [49,53,57,58]. It is important to note that all studies used multifactorial interventions [36,42,43,44,45,46,47,48,49,50,51,52,53,54,55,56,57,58,59] that focused not only on nutrition, but also on multiple components, including physical exercise (15/19) [36,42,43,44,45,46,47,48,50,52,53,54,56,57,59], counseling in health habits (3/19) [45,52,58], education (exercises, health habits, and medication) (5/19) [45,48,49,53,55], cognitive training [54], psychosocial support (2/19) [45,48], and medication adherence (2/19) [43,56] (Table 2). Consequently, it is challenging to isolate the impact of nutritional intervention on reported outcomes.

### 3.5. Main Findings

The majority of the studies evaluating effects on frailty (11/14) indicated statistically significant improvements in frailty scores after interventions were implemented [36,42,43,45,47,48,49,50,54,57,59]. Improvements were also noted in overall dietary intake outcomes and nutritional knowledge in three out of eight relevant studies [46,47,48]. Overall physical performance improved in all 12 relevant studies [36,44,46,47,49,53,54,55,56,57,58,59] and functional status improved in 9 out of 12 relevant studies [36,43,45,46,50,52,53,56,57]. Regarding psychosocial outcomes, all four studies showed improvements [36,46,47,48]. Additionally, cognitive function improved in three out of four studies [36,49,56] and overall quality of life improved in four out of six relevant studies [47,48,55,59]. Lastly, mortality only improved in one out of two studies [43]. Overall, significant improvements were noted not only in studies (5/19) with limited sample sizes (n = 13–32) but also in the majority of studies (13/19) [42,43,44,45,46,47,48,50,52,54,55,56,59] including larger sample sizes, with only one reporting inconsistent results [51].

#### 3.5.1. Frailty Scores

The results indicate that patients who were treated exclusively with nutritional interventions either via supplements [42,54] or in combination with counseling [47] exhibited a statistically significant improvement in frailty scores relative to patients who received usual care. The majority of the other studies that used multicomponent interventions with nutritional elements to evaluate frailty also supported these results [36,43,45,48,49,50]. Only one study showed that a comprehensive cardiac rehabilitation program including dietary guidance did not improve Kihon checklist scores used for frailty evaluation [53]; however, the authors acknowledge that detecting an improvement effect might have been difficult given the small sample size (n = 30).

#### 3.5.2. Dietary Intake Outcomes and Nutritional Knowledge

The study that exclusively used nutritional intervention (counseling and supplements) reported increased intake levels of total calories, protein, and carbohydrates [47]. Nutritional interventions, when integrated into a multicomponent strategy, were associated with improvements in caloric intake [46], legume intake [58], the Mini Nutritional Assessment [46,48], the Simplified Nutritional Appetite Questionnaire (SNAQ) [48], and adherence to the Mediterranean diet score [49,58]. A trend towards an improved nutritional status, as indicated by the Scored Patient-Generated Subjective Global Assessment (PG-SGA) score, was noted in a hospital-to-home exercise–nutrition self-managed intervention program at 3 months [36]. While the findings are encouraging, certain multicomponent interventions failed to improve scores in areas such as the Mediterranean diet knowledge test [49,58], the Geriatric Nutritional Risk Index (GNRI) [53], Controlling Nutritional Status [53], protein intake [55], and the Food Beliefs Survey’s five subscales [58], likely because the studies had a limited number of participants (15–44).

#### 3.5.3. Physical Performance Outcomes

The focus of our analysis was on five key physical performance outcomes in frail and pre-frail older adults undergoing nutritional interventions: body composition, gait speed, the Timed Up-and-Go test, handgrip strength, and the Short Physical Performance Battery.

Interventions involving nutritional supplementation or counseling via mobile apps do not appear to improve BMI, [54,58] nor do other programs where nutrition is a component of a multicomponent approach [55]. No changes were also observed in body composition (fat and skeletal mass) with a multicomponent intervention, namely Gamified Home-Based Cognitive–Nutritional (GAHOCON) [49]. BMI improved in only one study that used a complete geriatric assessment with dietary guidance [46].

Gait speed was improved by nutritional intervention through supplementation [54] or in conjunction with counseling [47]. Nutritional interventions in multicomponent studies have also shown improvements in the 6 min walk test (6MWT) [49,52,53], the 10 m walk test [55], and the number of daily steps [53].

However, no significant differences were observed in the six-minute walk test (6MWT) [47,57], the 10 m walk test [44], or 4 m gait speed [46] in other studies when nutritional interventions were incorporated into the multicomponent programs.

The Timed Up-and-Go Test (TUG) results from the only study included presented inconsistent findings. Adding nutritional supplements to the Otago Exercise Program, on the other hand, did not significantly affect the timed Up-and-Go test [44].

An exclusive nutrition intervention (counseling and supplements) significantly improved handgrip strength, upper and lower body flexibility, and lower extremity strength [47]; however, nutritional supplementation alone did not result in an improvement in knee extension strength [54]. In many studies, the incorporation of nutritional interventions in multicomponent programs led to improvements in handgrip strength (HGS) [36,44,47,56] and knee extension strength as well as the ratio of knee extension muscle strength to body weight [53]. However, this was not the case in some studies [49,55,57].

There was no improvement in the Short Physical Performance Battery (SPPB) score [58] after receiving nutritional counseling through a mobile application. Some research indicated that SPPB scores improved with multicomponent programs that involved nutritional interventions [36,46,50,56], whereas other studies found no such effect [44,52,53,57].

#### 3.5.4. Functional Outcome Measures

Nutritional supplementation alone did not yield an improvement in dependency related to instrumental activities of daily living (IADL) and activities of daily living (ADL) [54]. Multicomponent interventions, including nutritional strategies, decreased the risk of functional decline (Barthel index and Lawton scale scores [43]), enhanced Berg Balance Scale performance [44], improved disability, as measured by the World Health Organization Disability Assessment Schedule (WHODAS) [52], and increased functional reach and unipodal stance [56], sit-to-stand functioning (30 s chair stand test), and dynamic balance (four-square step test) [55], and they marginally improved Katz ADL [48]. In contrast, no significant improvements were noted in the Barthel index (for basic daily activities) [45,46], Lawton instrumental activities of daily living (IADL) [46], Lawton and Brody score [45], repeated chair stand test [44], risk of functional decline assessment [36], Lung Transplant Valued Life Activity (LT-VLA), and Duke Activity Status Index (DASI) [57].

#### 3.5.5. Psychosocial Outcomes

The Geriatric Depression Scale scores showed improvement with nutrition intervention alone (counseling and supplements) [36,47], as well as when combined with other multicomponent interventions [46].

#### 3.5.6. Cognitive Function

When it comes to cognitive function, multidisciplinary interventions that include nutritional components appear to be beneficial, based on results from tests like the Mini-Mental State Examination (MMS) [36], Montreal Cognitive Assessment (MoCA) [49], and other memory performance metrics [56]. On the contrary the MMS scores did not improve with a comprehensive geriatric assessment (CGA) that included nutritional counseling [46], and the Fuld Object Memory Evaluation (FOME) remained unchanged following the Gamified Home-Based Cognitive–Nutritional (GAHOCON) intervention [49].

#### 3.5.7. Quality of Life

Improvement in the 12-Item Short-Form Health Survey mental component summary was seen with an exclusive nutritional intervention that included counseling and supplementation [47]. Studies on multicomponent interventions, including nutritional components, indicated improvements across most WHOQOL-BREF questionnaire domains (physical health, mental health, social relations, and environment) [48], HRQoL (EQ-5D-5L index score), and self-rated health on the visual analog scale of the EQ-5D-5L [55]; however, other research did not find similar effects on health-related quality of life (EQ-5D-5L) [36,52].

#### 3.5.8. Mortality

Mortality was evaluated in merely two studies, with a comparatively short follow-up duration. Recommendations on nutrition as part of a coordinated intervention decreased mortality three months after discharge [43], but another program with nutritional assessment and counseling did not improve mortality at 180 days [51].

## 4. Discussion

The present scoping review aimed to highlight nutritional interventions that could positively benefit older patients with frailty and pre-frailty. The results of the studies included in our review indicate that pre-frailty and frailty, particularly in their early stages, might be improved or even reversed, with nutritional interventions possibly playing a crucial role. In addition, the results of the included studies report that patients who underwent nutritional interventions exclusively or within multicomponent interventions displayed a statistically significant improvement in physical performance and functional outcomes, depressive symptoms, cognitive function, and quality of life across several studies. Nonetheless, the interpretation of these findings requires caution due to the multifactorial nature of most interventions and the heterogeneity in frailty tools used in the included studies (e.g., Fried’s vs. CHS). Given the scope of this review, which excludes data pooling for quantitative analysis, it is difficult to isolate the effects of nutritional interventions in cases of frailty that are defined in a consistent way.

A major finding of the present study was that nutrition-based educational interventions (i.e., educational sessions, presentations, using mobile health apps, or game-like home training) and nutritional counseling interventions could improve nutritional knowledge, adherence to diet, physical activity (i.e., enable patients to maintain their daily activities), nutritional status, and overall quality of life. Interestingly, a review on nutrition interventions for older adults with frailty found that education, including counseling, led to small but noticeable improvements in physical performance and mobility [60]. Moreover, a study conducted on lung cancer patients with frailty found that dietary quality-focused nutrition education significantly improved frailty, nutritional status, and overall quality of life [61]. In addition, another meta-analysis reported that nutritional management interventions improved body weight and gait speed in frail and pre- frail older patients [21]. However, another meta-analysis emphasized that nutrition-based interventions alone are not enough to improve frailty [34]. This highlights that nutrition-based educational interventions could be combined with other interventions (i.e., interventions that improve physical activity) to have the biggest impact on older patients with frailty [62].

Another significant finding of the present review was that protein supplementation, whether through branched-chain amino acids or personalized plans, was positively associated with enhanced handgrip strength, walking speed, and overall nutritional well-being, while simultaneously mitigating frailty. This aligns with a meta-analysis that concluded that nutritional interventions, primarily protein and vitamin D supplements, significantly improved grip strength, calf circumference, and performance on the timed Up-and-Go test, with moderate to high certainty for physical outcomes in frail older adults [34]. Another meta-analysis on nutritional management further supported these findings, reporting better outcomes in areas such as frailty reversal when protein was part of comprehensive strategies, although standalone supplementation had inconsistent effects on psychological aspects [62]. This indicates that protein supplementation could be a useful approach, especially for malnourished frail older patients. Nevertheless, despite the potential positive effects of protein supplements on frailty, it should be noted that excessive protein intake can be harmful [63]. Therefore, when recommending an increase in protein intake, healthcare professionals should consider the individual characteristics of patients [63]. Furthermore, determining individual optimal protein intake necessitates further research to consider variations in patients’ baseline nutritional status.

The studies included in our review also report that interventions that combined physical activity promotion with protein supplementation could yield greater improvements in frailty, and even in some cases reversed it. Moreover, physical activity with nutritional educational/counseling interventions also seems to improve frailty. Furthermore, 4/19 studies incorporated long-term follow-up interventions (ranging from 12 to 24 months) [45,50,54,56], which continued to demonstrate positive outcomes, despite barriers such as incomplete adherence across all interventions post implementation. However, studies with multiple components, short follow-up time, and lack of quality appraisal make it difficult to examine the impact of each component separately. Nevertheless, these findings align with a systematic review and meta-analysis that analyzed multicomponent interventional studies (including nutritional components) and found that combined interventions are better than nutrition-based interventions alone [60]. Similarly, another meta-analysis on combined exercise and nutrition in hospitalized pre-frail/frail patients reported greater reductions in frailty scores and improvements in short physical performance scores, particularly in chair stand tests, although results varied for activities of daily living [31]. Importantly, recent studies consistently report that multifaceted interventions have the highest benefits for older patients with frailty, including physical and mental health [31,33,60,64,65]. A potential explanation for this could be that these interventions have a combined effect, for example nutrition supporting muscle recovery after exercise [31]; while this could be the case, future studies could focus on developing standardized frailty assessment tools that are tailored for various settings and types of frailty in order to improve the comparability of results.

Our findings have numerous implications for clinical practice, research, and public health. First, clinically, incorporating multifaceted approaches, combining nutrition (e.g., protein supplements to boost grip strength and overall nutrition), physical activity, and nutrition education, into geriatric care could improve patient adherence, physical performance, and mental well-being. This integrated approach could also yield better results than isolated strategies (single intervention). To that end, the use of technology-enhanced delivery has been highlighted as an emerging domain within the literature; this may have important implications for scalability and accessibility, especially in resource-constrained settings. Second, future studies could address gaps in long-term micronutrient effectiveness and the underlying mechanisms through RCTs across diverse populations, including those with existing health conditions. Further exploration of microbiota modulation, amino acids, and cognitive training could assist in refining multidomain interventions that may reduce frailty risk. Moreover, future studies could utilize objective nutritional status assessment and associated biomarker signals in older patients, thereby enhancing the selection and monitoring of nutrition interventions [66,67]. Finally, public health initiatives, such as policies that promote essential nutrients like protein and vitamin D, coupled with community-based education programs could assist in preventing the onset of frailty, lower healthcare costs by reducing hospitalizations, and promote healthier aging globally by encouraging early and sustainable lifestyle changes.

## 5. Limitations

The present scoping review, while contributing significantly to the existing body of knowledge by offering a comprehensive look at nutritional interventions for older adults with frailty and pre-frailty, is subject to certain limitations that should be taken into consideration. First, the definition and assessment of frailty is subject to methodological variations and poses challenges to comparisons among studies. Second, the intervention outcomes frequently focus on a specific aspect of the frailty phenotype, as opposed to overall frailty. Third, many studies have incorporated multifaceted approaches, such as combined exercise and nutrition plans, which complicates the isolation of nutrition’s role. Fourth, the use of supplements and foods, which typically include various nutrients, makes it difficult to understand the impacts of each component. Fifth, the present review did not include a quality assessment of the articles, and thus the conclusions were based on a descriptive synthesis, without further statistical analysis. Sixth, the protocol was retrospectively registered after the search, which may introduce selection bias. Nevertheless, no deviations from the initial plan were made. Regarding potential selection bias, the language restriction (English only) and the date restriction (last 10 years) of the studies excluded non-English or older studies; however, this was carried out to provide the most accessible and up-to-date studies. Seventh, the search of the literature encompassed two databases (MEDLINE and Scopus), which may have omitted the inclusion of studies from other sources or gray literature. Lastly, the scoping nature of this study prioritized breadth over depth, lacking additional statistical analyses to quantify effect sizes or explore subgroups like gender or comorbidities.

## 6. Conclusions

Our findings indicate that nutritional interventions either alone or as part of multifactorial interventions appear to have potential in improving the quality of life of older patients with frailty. However, the heterogeneity in intervention design, frailty definitions, and outcome measures significantly limited the studies’ comparability. Nevertheless, these findings indicate that a multifaceted approach combining nutrition, physical activity, and nutrition education in geriatric care could improve patient outcomes. Future research should employ standardized frailty criteria and optimal dietary interventions with extended intervention durations to strengthen the evidence and guide community-based initiatives, ultimately aiming to prevent and manage frailty, reduce healthcare expenditures, and promote healthier aging.

## Figures and Tables

**Table 1:** Characteristics of included studies.

Author/Year (Ref.)	Study Design	Country	Setting	Population	Nutritional Intervention	Control	Duration of Intervention (Follow Up)	Outcomes
Caldo-Silva et al., 2023 [42]	Non-RCT	Portugal	Nursing homes	n = 188 frail	Branched-chain amino acid (BCAA) supplementation	Νon-exercise/no-supplementatin	40 weeks (40 weeks)	Physical frailty status Hematological biomarkers
Checa-López et al., 2023 [43]	RCT	Spain, Italy, United Kingdom	Hospitals	n = 821 frail	Nutritional counselling	Usual care	Hospitalization time (3 months after discharge)	Functional status Institutionalization Mortality Disability Visits to the ER Hospital readmissions Frailty status
García-Gollarte et al., 2023 [44]	RCT	Spain	Long-term care facilities	n = 111 frail	Oral nutritional supplementation	Usual care	6 months (6 months)	Functional status Physical performance Waist circumference BMI
González-Mariscal et al., 2025 [45]	RCT	Spain	Community Health Centers	n = 199 frail/pre-frail	Nutritional education	Usual care	6 months (12 months)	Frailty status Functional status
Han et al., 2023 [36]	RCT	Australia	Hospital	n = 32 frail/pre-frail	Individualized nutrition care plan	Usual care	Hospitalization time and 3 months post discharge (6 months)	Program adherence Frailty status Functional status Physical performance Nutritional status Cognitive function Mental health Quality of life Unplanned readmissions
Hsiao et al., 2024 [46]	Cohort	Taiwan	Geriatric Clinic	n = 100 frail/pre-frail	Nutritional counseling	N/A	2 months (2 months)	Nutritional status Frailty status Functional status Physical performance BMI Cognitive function Mental health
Hsieh et al., 2019 [47]	RCT	Taiwan	Hospital	n = 319 frail/pre-frail	Nutritional counseling and nutritional supplementation	Usual care	3 months (6 months)	Frailty status Quality of life Physical performance Mental health
Kasa et al., 2024 [48]	Quasi-experimental	Ethiopia	Community	n = 68 frail	Nutritional education	-	6 months (9 months)	Frailty status Functional status Nutritional status Mental health Quality of life
Kwan et al., 2024 [49]	RCT	China	Community	n = 25 frail	Nutritional education and Gamified Home-Based Cognitive–Nutritional intervention	Only health and nutritional education	12 weeks (13 weeks)	MD knowledge and adherence Cognitive function Physical frailty status Physical performance Body composition
Laosa et al., 2025 [50]	RCT	Spain, Czech Rep, Italy, France, Germany	Community	n = 298 frail/pre-frail	Nutritional education	Usual care	4 months (24 months)	Frailty status Functional status Physical performance
Lembeck et al., 2019 [51]	RCT	Denmark	Patients after hospital discharge	n = 537 frail	Nutritional counseling	Usual care	2 days (6 months)	Readmissions Visits to GPs Mortality
McIsaac et al., 2022 [52]	RCT	Canada	Perioperative care patients	n = 204 frail	Nutritional education	Administration of Canada’s Food Guide	4 weeks (30 days after hospital discharge)	Functional status Physical performance Quality of life Readmissions In-hospital complications and length of stay Hospitalization costs
Nagatomi et al., 2022 [53]	RCT	Japan	Outpatients with chronic heart failure	n = 30 frail	Nutritional education	Usual care	3 months (3 months)	Physical frailty Functional status Physical performance Cardiac failure status Nutritional status Hematological biomarkers
Ng et al., 2015 [54]	RCT	Singapore	Community	n = 246 frail/pre-frail	Nutritional supplementation	Usual care	6 months (12 months)	Frailty status BMI Activities of daily living Hospitalizations Physical performance
Rodrigues et al., 2022 [55]	RCT	Canada	Nursing homes/assisted living, community	n = 44 frail/pre- frail	Nutritional education	Usual care	3 week (16 weeks)	Quality of life Physical performance Total calorie consumption Health care resource utilization and costs
Romera-Liebana et al., 2018 [56]	RCT	Spain	Primary Health Care Centers users	n = 352 frail/pre-frail	Intake of hyperproteic nutritional shakes	Usual care	12 weeks (18 months)	Frailty status Physical performance Functional status Cognitive function Neuropsychological performance Medication number
Singer et al., 2018 [57]	Non-RCT	USA	Lung transplant candidates	n = 13 frail	Nutritional counseling	Usual care	8 weeks (8 weeks)	Frailty status Functional status Physical performance
Su et al., 2023 [58]	RCT	USA	Community	n = 15 frail/pre- frail	Nutritional counseling via mobile app	Online healthy eating materials	3 months (3 months)	MD adherence Insulin resistance Physical performance Functional status Eating habbits Behavior change Metabolic panel, CRP
Yuri et al., 2016 [59]	non-RCT	Japan	Community	n = 143 frail	Nutritional education	Standard preventive care	3 months (9 months)	Physical performance Frailty status Quality of life Life goals

Abbreviations: RCT: Randomized controlled trial; ER: Emergency Room; BMI: body mass index; GP: General Practitioner; MD: Mediterranean Diet; CRP: C-reactive protein.

**Table 2:** Main findings of the studies.

Author/Year (Ref.)	Frailty Diagnostic Tool/Criteria Used	Measurement of Outcomes	Intervention	Main Results
Caldo-Silva et al., 2023 [42]	Fried’s Frailty Phenotype (FP) Scale	Hematological biomarkers	Four experimental groups: Multicomponent exercise program (MEP) plus branched-chain amino acids (BCAA); MEP only; BCCA only; and the non-exercise/no-supplementation control group.	BCAAs *, especially when combined with MEP, lead to improvements in hematological biomarkers (MCH and MCHC), as well as in physical frailty (reduction in the frailty index).
Checa-López et al., 2023 [43]	Fried’s Frailty Phenotype (FP) Scale FRAIL scale	Barthel index for functional status. Institutionalization (yes/no). Mortality (yes/no). Lawton scale for independent status. Visits to the ER (yes/no). Hospital readmissions (yes/no).	Two groups: Intervention group with a comprehensive geriatric assessment and a coordinated intervention (recommendations on polypharmacy, delirium, falls, nutrition, physical exercise) plus a discharge plan; usual care in control group.	The intervention reduced the risk of functional deterioration (Barthel index and Lawton scale scores) and mortality, reduced readmissions at 3 months, and improved frailty in the cardiology setting.
García-Gollarte et al., 2023 [44]	Fried’s Frailty Phenotype (FP) Scale	Timed Up-and-Go test (TUG), Berg Balance Scale (BBS), 6MWT, 10MWT, Short Physical Performance Battery (SPPB), repeated chair stand test (STS-5) for functional performance. Handgrip strength (HGS) with dynamometer. Barthel index for functional status.	Three groups: Multicomponent Otago Exercise Program (OEP); OEP with oral nutritional supplementation (OEP+N); control group.	Adding a nutritional supplementation to the OEP improved the Berg Balance Scale and handgrip strength.
González-Mariscal et al., 2025 [45]	Fried’s Frailty Phenotype (FP) Scale	Barthel index for functional status. Lawton scale and Brody scale for instrumental activities of daily living.	Two groups: Intervention group with health education program consisting of four group sessions (first month) and six follow-up calls (the following five months) focused on frailty description and its impact on health, guidelines for physical activity, promoting healthy dietary habits, cognitive training, psychological and social well-being; control group.	The intervention group showed higher scores that were sustained over time (meaning a higher functionality) in the Barthel index and Lawton and Brody scale scores and lower points (meaning a lower degree of frailty) in the frailty score than the control group.
Han et al., 2023 [36]	Edmonton Frail Scale (EFS)	Short Physical Performance Battery (SPPB) for physical performance. Handgrip strength (HGS) with dynamometer. Scored Patient-Generated Subjective Global Assessment (PG-SGA) for nutritional status. Mini-Mental State Examination (MMSE). EQ-5D-5L (QoL). Geriatric Depression Scale (GDS). Hospital Admission Risk Profile. Hospital outcomes.	Two groups: Intervention group with individualized exercise and nutrition care plan created by the research dietitian and physiotherapist while admitted that continued for 3 months post discharge through an ambulatory service in the form of four home visits and four telephone calls; control group.	Participants in the intervention group had significantly greater improvement in EFS, overall SPPB score, Mini-Mental State Examination, handgrip strength, and Geriatric Depression Scale compared to the control.
Hsiao et al., 2024 [46]	Clinical Frailty Scale (CFS)	Body mass index (BMI). Mini Nutritional Assessment-Short Form (MNA-SF) for nutritional risk. Mini-Mental State Examination (MMSE) for cognitive function. Geriatric Depression Scale-15 (GDS-15). Short Physical Performance Battery (SPPB) for physical performance.	One group: Guidance-based frailty intervention model for holistic approach to frailty management (comprehensive geriatric assessments, case management, nutritional counseling by dietitian, physical activity, monitoring and follow-up via telephone calls).	The intervention significantly improved mood (GDS-15), physical performance (SPPB), BMI and nutritional status (caloric intake and MNA-SF).
Hsieh et al., 2019 [47]	Fried’s Frailty Phenotype (FP) Scale	Handgrip strength, 10 m gait speed, upper body flexibility, lower body flexibility, lower extremity strength, Short-Form Health Survey mental component summary (SF-12 MCS), Geriatric Depression Scale (GDS).	Four groups: Exercise, nutrition (food supplements and counseling), exercise plus nutrition (food supplements and counseling), and control group.	The nutritional intervention significantly increased the intake levels of total calories, protein, and carbohydrate and improved frailty score, handgrip strength, 10 m gait speed, upper body flexibility, lower body flexibility, and lower extremity strength, mood, and SF-12 MCS score.
Kasa et al., 2024 [48]	Tilburg Frailty Indicator Amharic Version (TFI-AM)	Mini Nutritional Assessment (MNA) and Simplified Nutritional Appetite Questionnaire (SNAQ) for nutritional status. Geriatric Depression Scale (GDS-15). Katz-ADL for activities in daily living. WHOQOL-BREF for quality of life.	One group: Based on the Integral Conceptual Model (ICMF), a nurse-led education intervention (handbook culturally contextualized to frailty management, six independent, interconnected education sessions on aging and age-related changes, healthy nutrition, physical activity, mental health, social interaction and support, and an overall discussion).	The intervention significantly improved frailty MNA, SNAQ, GDS-15, and most components of the WHOQOL-BREF.
Kwan et al., 2024 [49]	Fried’s Frailty Phenotype (FP) Scale	Mediterranean diet knowledge and adherence. Cognitive function (Montreal Cognitive Assessment-MoCA). 6 min walk test (6MWT) for walking speed. Body composition. Fuld Object Memory Evaluation (FOME) for memory. Dynamometer for handgrip strength.	Gamified Home-Based Cognitive–Nutritional (GAHOCON). Two groups: Intervention group with 4 weeks of center-based training and home-based (health education) Mediterranean diet followed by 8 weeks of home-based training (GAHOCON); control group received only the 4 weeks of center-based training and 8 weeks of self-revision of health educational materials at home.	Significant improvements were observed in the intervention group in Mediterranean diet adherence score, cognitive function, frailty, and walking speed compared to the control group.
Laosa et al., 2025 [50]	Fried’s Frailty Phenotype (FP) Scale	Short Physical Performance Battery (SPPB).	Multi-modal intervention (MIDFRAIL). Two groups: Intervention group with individualized 16-week resistance exercise program, nutritional educational sessions, optimization of diabetes care; control group usual care.	The MIDFRAIL intervention group improved frailty status and physical performance (SPPB) at long-term follow-up in older people with T2D.
Lembeck et al., 2019 [51]	Frailty model	Mortality	Two groups: Intervention group (assessment of cognitive skills, medicine, nutrition, home environment, mobility, level of functioning and future appointments in the health care sector) and then individualized counseling, home care, and rehabilitation in the municipality; control group usual care.	The intervention and control groups had similar mortality at 8, 30, and 180 days and the survival curve showed a similar pattern.
McIsaac et al., 2022 [52]	Clinical Frailty Scale (CFS)	6 min walk test (6MWT). Short Physical Performance Battery (SPPB). EQ-5D-5L for quality of life. World Health Organization Disability Assessment Schedule (WHODAS) for disability. Postoperative Morbidity Survey (POMS) for in-hospital complications. Length of index hospitalization.	Two groups: Intervention group (4 weeks before surgery) with home-based body exercise training (exercise prehabilitation), healthy eating before surgery guide; control group (30 days after surgey) received the WHO Global Recommendations for Physical Activity for Health, Canada’s Food Guide, and a pedometer support.	Adherent to prescribed exercises showed significant improvements in 6 min walk test and disability.
Nagatomi et al., 2022 [53]	Japanese Cardiovascular Health Study (J-CHS) scale Kihon checklist	New York Heart Association functional classification II–III for chronic HF. 6 min walk test (6MWT) for walk distance. Brain natriuretic peptide (BNP). Kansas City Cardiomyopathy Questionnaire (KCCQ) for health-related (HR) QoL. Short Physical Performance Battery (SPPB) for balance. Geriatric Nutritional Risk Index (GNRI) for nutritional information.	Two groups: Comprehensive home-based cardiac rehabilitation (HBCR) program including continuous evaluation and determination of appropriate exercise intensity and dietary guidance for a period of 3 months; control group usual medical care.	The intervention group showed a significantly improved 6 min walking distance (6MWD), knee extension strength, and knee extension muscle strength-to-body weight ratio as well as number of steps.
Ng et al., 2015 [54]	CHS physical frailty criteria	Knee strength. Body mass index (BMI). 6 m fast gait speed test. SF-12 scale. Longitudinal Aging Physical Activity Questionnaire. Instrumental activities of daily living (IADL) and activities of daily living (ADL) dependency.	Five groups: Interventions group with nutritional supplementation, cognitive training, physical training, combination training; control group usual care.	Nutritional supplementation reduced frailty, and, in particular,, increased the level of physical activity.
Rodrigues et al., 2022 [55]	CHS physical frailty criteria	Dynamometer for handgrip strength. 30 s chair stand test. Four-square step test (FSST). 10 m walk test. EuroQol Group 5 Dimension 5 Level (EQ-5D-5L) for HRQoL. Automated Self-Administered 24 h (ASA24) Dietary Assessment Tool.	MoveStrong Program. Two groups: Intervention group with 16 exercise physiologist-led hour-long sessions and two dietitian-led hour-long nutrition sessions and adequate protein intake; control group usual care.	Intervention improved gait speed (10 m walk test), sit-to-stand functioning (30 s chair stand test), dynamic balance (four-square step test) and HRQoL (EQ-5D-5L index score).
Romera-Liebana et al., 2018 [56]	Fried’s Frailty Phenotype (FP) Scale	Short Physical Performance Battery (SPPB). Functional Reach Test. Dynamometer for handgrip strength. Unipodal Station Test, which measures balance. Short- and Medium-Term Verbal Memory.	Two groups: Intervention group with structured physical exercise, hyperproteic nutritional shake, memory workshops, medication review; control group usual care.	After the intervention, there were significant improvements in Short Physical Performance Battery score, handgrip strength, functional reach, unipodal station, and all memory performance measures.
Singer et al., 2018 [57]	Fried’s Frailty Phenotype (FP) Scale	Short Physical Performance Battery (SPPB), 6 min walk test (6MWT) for physical performance. Fitbit device for weekly step count. Dynamometer for handgrip strength. Lung Transplant Valued Life Activity (LT-VLA) and Duke Activity Status Index (DASI),	Two groups: Intervention group with home-based exercise and nutrition components via mobile device application (platform Aidcube™) and Fitbit® for physical activity monitoring in real time; control group usual care.	A trend towards improvement in SPPB and frailty scores from before to after the intervention and some patients transitioned from frail to not-frail status.
Su et al., 2023 [58]	CHS physical frailty criteria	Body mass index (BMI). Food frequency questionnaire (FFQ) Mediterranean Diet Index. Mediterranean Diet Nutrition Knowledge (MDNK). Food Beliefs Survey 5 subscales (Positive and Negative outcome expectations, Self-efficacy, Self-regulation, Social support). Short Physical Performance Battery (SPPB).	Two groups: Intervention group with mobile app “Olitor” providing feedback on food choices, personalized recipes, and in-app messaging to improve adherence to the Mediterranean diet; control group received referrals to healthy eating materials.	Nutritional counseling via mobile app improved the Mediterranean diet adherence score and legume intake.
Yuri et al., 2016 [59]	Kihon Checklist	5-point answer scale for self-rated health. Questionnaires for life goals. Canadian Occupational Performance Measure (COPM) for physical performance. Medical Outcomes Study (MOS-20) and Older People’s Quality Of Life (OPQOL) questionnaire for quality of life.	Two groups: Intervention group with Life Goal-Setting Technique (LGST) support plus standard preventive care (SPCP) program including physical exercise classes, oral care and nutrition education; control group only standard preventive care (SPCP) program.	The intervention with life goal-setting support improved frailty status and self-rated quality of life, grip strength, sit and reach test and timed Up-and-Go test.

* BCAAs: branched-chain amino acids, MCH: Mean Cell Hemoglobin, MCHC: Mean Cell Hemoglobin Concentration, MEP: Multicomponent exercise intervention.

## Data Availability

No new data were created or analyzed in this study.

## Supplementary Materials

The following supporting information can be downloaded at: https://www.mdpi.com/article/10.3390/jcm15020604/s1, Table S1: Preferred Reporting Items for Systematic reviews and Meta-Analyses extension for Scoping Reviews (PRISMA-ScR) Checklist.
